# Long-latency auditory evoked potential in children with stuttering

**DOI:** 10.31744/einstein_journal/2020AO5225

**Published:** 2020-06-03

**Authors:** Gislaine Machado Jerônimo, Ana Paula Rigatti Scherer, Pricila Sleifer

**Affiliations:** 1 Universidade Federal do Rio Grande do Sul Porto AlegreRS Brazil Universidade Federal do Rio Grande do Sul, Porto Alegre, RS, Brazil.

**Keywords:** Electrophysiology, Evoked potentials, Hearing, Stuttering, Child

## Abstract

**Objective:**

To analyze the latency and the amplitude values of Mismatch Negativity and P300 cognitive potential in children with stuttering, with no auditory complaints, with auditory thresholds within the normality range, comparing them to the findings of a Control Group.

**Methods:**

A cross-sectional study involving 50 children of both sexes, 15 with stuttering and 35 without stuttering, aged 6 to 11 years, with no diagnosis of ear pathology or other diseases. All children were submitted to peripheral audiological evaluation (meatoscopy, pure tone testing, speech audiometry, and acoustic immittance measures) and a central audiological evaluation (investigation of the Mismatch Negativity and P300 cognitive potential). For the evaluation of fluency, all children with stuttering had a specific history taken and were video recorded in a spontaneous speech. Afterwards, the transcription was done, followed by speech analysis to classify children according the severity of stuttering.

**Results:**

There was a significant difference in the latencies of Mismatch Negativity and P300 cognitive potential, as well as in the amplitude of Mismatch Negativity.

**Conclusion:**

There was a significant delay in the latencies of Mismatch Negativity and P300 cognitive potential, as well as increase in the amplitude of the Mismatch Negativity in children with stuttering when compared to children in the Control Group. Changes in the morphology of the waves were found in the Stuttering Group.

## INTRODUCTION

Long-latency auditory evoked potentials (LLAEP) are used in cognitive investigation, especially the P300 cognitive potential, which is endogenous and shows bioelectrical responses that run through the thalamus and cortex.^(1,2)^ Mismatch Negativity (MMN), another endogenous potential, is an LLAEP that reflects the electrical brain response of processing skills, discrimination and auditory memory.^(3)^ Mismatch Negativity is a potential that detects changes in discernible auditory stimulus, and it is generated in the frontal cortex, thalamus and hippocampus.^(3,4,5)^

The main characteristic of MMN is that it registers without the influence of the individual’s attention or demands from tasks, which makes it efficient for clinical studies in different populations.^(6)^The P300 potential is generated voluntarily and actively in the resolution of a specific task: it evaluates perception, attention and auditory memory skills. It is generated in the hippocampus, the mesencephalic reticular formation, the medial thallus, the pre-frontal cortex and parietotemporal association areas.^(1)^

Regarding children, there are studies with MMN^(3,7,8)^ in populations with autistic spectrum disorder, auditory processing disorders, cleft lip and palate, prematurity, and language development disorder. Most articles are about dyslexia, and MMN is underexplored in individuals with stuttering.^(8)^ In the Brazilian population, only one published study was found that investigated P300 in children who stutter.^(9)^ Up till now, there are no studies with children using both techniques, MMN and P300, concomitantly.

Stuttering is a fluency disorder, which involves alterations in speech flow. They are atypical and involuntary interruptions, hinder smooth and fluid speech, and are characterized by repetitions, prolongation and blocks. It has a 1% prevalence in the global adult population and affect up to 5% of children.^(10,11)^ Stuttering that develops during childhood is called persistent developmental stuttering (PDS) and affects 5% of children^(12,13)^

Current theories use a combination of genetic, neurological, motor, linguistic and environmental factors to explain the etiology of PDS.^(14)^ Neuroimaging shows that children who stutter present neuroanatomical and functional differences.^(10)^

Auditory aspects may be impaired and could interfere in speech fluency.^(11)^ Therefore, investigating LLAEP in relation to stuttering is very important because, through an objective evaluation, it might result in a better understanding of the factors that interfere in speech fluency and help with therapeutic rehabilitation techniques.

## OBJECTIVE

To analyze the latency and amplitude values of long-latency auditory evoked potentials in children with stuttering and without auditory complaints, with auditory thresholds within normality and compare the results to a Control Group.

## METHODS

This is an observational, cross-sectional, comparative study, contemporary and individual, carried out in 2017. The study was approved by the Research Ethics Committee of the *Instituto de Psicologia da Universidade Federal do Rio Grande do Sul* (UFRGS), protocol number 2011039. The convenience sample included children of both sexes, aged between 6 and 11 years, divided into two groups: Study Group with Developmental Stuttering (SG) with children with mild to severe stuttering, and Control Group (CG) with children with normal development.

Information about age; sex; manual preference; level of education; language, social, neural and hearing impairment; and about patients undergoing speech therapy or not were obtained through general patient’s history. Exclusion criteria included hearing, language and learning complaints; inability to count from 1 to 50; any type of hearing loss; otorhinolaryngological evaluation showing alterations; psychiatric syndromes or alterations; having undergone speech therapy for stuttering; and not having done the procedures or concluded exams for any reason. All the children underwent peripheral auditory assessment and central electrophysiological assessment.

The SG was previously triaged by two speech therapists. The diagnosis of stuttering was verified and confirmed by speech therapists with experience in the area. The Kappa measurement was used for diagnosis agreement analysis.

For the audiological evaluation, patient history was taken, external acoustic meatuses were inspected, and pure tone audiometry threshold, speech audiometry, and immittance test were performed. The research about MMN and P300 was done using the equipment Masbe ATC Plus by Contronic^®^, with inspection earphones 3A and silver electrodes. Electrical impedance was under 5Ω in each derivation, and the difference between the three electrodes was more than 2Ω. After verifying the impedance, the electroencephalogram scan was performed.

The parameters used for the MMN study were auditory stimuli presented in monoaural mode, with a frequency of 1,000Hz (50 cycles), for the frequent stimulus, and 2,000Hz (50 cycles), for the rare stimulus, with an intensity of 70 dBNA for both. The presentation rate was of 1.8 pulses per second (pps). The average was 2,000 stimuli and the paradigm was 90/10, with alternated polarity. For the acquisition, the full scale was 200µV; a 1Hz high-pass filter; 20Hz low-pass filter; Notch:YES; 500ms temporal window; and tracing amplitude up to 7.5µV. During this process, the individuals were conditioned to watch an interesting silent video on a tablet to distract them from the auditory stimuli presented. Before the exam, the children were instructed to pay attention to the video.

For the P300 study, binaural stimuli with 80 dBNA intensity were used for both ears. The frequency was 1,000Hz with 50 cycles of duration and 20% rise and decay time, with trapezoidal envelope. The frequency for the rare stimuli was of 2,000Hz tone burst, with 100 cycles of duration with 20% rise and decay time with trapezoidal envelope, presented in oddball paradigm, with an occurrence probability of 80% and 20%, respectively. The stimuli were presented at a rate of 0.8pps. In the acquisition, the full scale was of 200µV; 01Hz high-pass filter; 20Hz low-pass filter; Notch: YES; and a 1,000ms temporal window was used. During this process, the children had to pay attention to the frequent and rare auditory stimuli presented, but only had to count the rare ones. The latency of P300 was marked at the point of maximum amplitude of the wave, and the analysis was done through the resulting wave.

The exams were performed twice. In order to verify the agreement of the analyses of MMN and P300, Kappa statistics were used. The results were organized in descriptive statistical form. The Kolmogorov-Smirnov test was used to evaluate data normality. To compare the ears in relation to latency and amplitude results, the Student *t* test was used. Significance level was set at 5% (p<0.05), and the analyses were done through the software (SPSS) for Windows, version 17.0.

## RESULTS

Fifteen children with different stuttering severity levels were selected: mild to moderate (n=4); moderate (n=6); moderate to severe (n=2); severe (n=1); and severe to extremely severe (n=2). In total, 50 children participated effectively in the study. Table 1 shows the sample characterization.

**Table 1:** Sample characterization

Variables	SG (n=15)	CG (n=35)
Sex		
Male	12 (80)	20 (57.14)
Female	3 (20)	15 (42.86)
Manual Preference		
Right-handed	8 (53.3)	31 (88.6)
Left-handed	7 (46.7)	3 (8.6)
Ambidextrous	0	1 (2.9)
Age, minimum/maximum (6-11)	8.40±1.80	9.29±1.52

Results are expressed in n (%) or mean±standard deviation.

SG: Study Group with Developmental Stuttering; CG: Control Group.

There was excellent agreement among the raters in the severity analysis of stuttering (Kappa 0.82) and in the analysis of the components of the LLAEP MMN and P300 (Kappa 0.89). According to the interclass correlation coefficient, 0.76 was found for MMN and 0.85 for P300, with a nearly perfect correlation.

All children responded to the MMN evaluation. Means and standard deviations of the latency and amplitude for the right ear (RE) and left ear (LE) are shown in table 2.

**Table 2:** Evaluation of Mismatch Negativity for latency and amplitude

Ear	SG (n=15)		CG (n=35)		p value*
	
	n	Mean±standard deviation	n	Mean±standard deviation	
Right					
Latency MMN	15	332.01±77.65	35	185.24±43.57	<0.001^*^
Amplitude MMN	15	8.11±3.28	35	5.25±1.61	<0.001^*^
Left					
Latency MMN	15	330.66±81.21	35	182.24±37.80	<0.001^*^
Amplitude MMN	15	7.75±3.30	35	5.65±2.21	0.011^*^

* Student’s *t* test; p≤0.05 significant.

SG: Study Group with Developmental Stuttering; CG: Control Group; MMN: Mismatch Negativity.

In the P300 study, there was a response from 14 of the children with stuttering – only one child did not respond. The differences in performance observed between the groups are shown in table 3.

**Table 3:** Results obtained in the P300 evaluation for latency and amplitude

Variables	SG (n=14)		CG (n=35)		p value*
	
	n	Mean±standard deviation	n	Mean±standard deviation	
Latency P300	14	697.19±142.84	35	308.17±18.81	<0.001^*^
Amplitude P300	14	11.70±3.89	35	13.53±4.85	0.216

* Student’s *t* test; p≤0.05 significant.

SG: Study Group with Developmental Stuttering.

Table 4 shows the means and standard deviations of latency and amplitude of the LLAEP MMN and P300 of left-handed children in relation to right-handed children.

**Table 4:** Comparison of latency and amplitude of Mismatch Negativity and P300, according to the manual preference of the children in the Study Group with Developmental Stuttering

Variables	Right-handed		Left-handed		p value*
	
	n	Mean±standard deviation	n	Mean±standard deviation	
MMN right ear					
Latency	8	322.40±85.83	7	342.99±72.17	0.880
Amplitude	8	7.99±3.18	7	8.26±3.63	0.627
MMN left ear					
Latency	8	314.04±74.85	7	349.65±89.84	0.065
Amplitude	8	6.30±2.62	7	9.42±3.37	0.417
P300					
Latency	8	706.30±147.12	6	685.05±149.80	0.997
Amplitude	8	11.71±3.14	6	11.71±5.06	0.795

* Student’s *t* test; p≤0.05 significant.

MMN: Mismatch Negativity.

## DISCUSSION

The analyzed sample is compatible to the number of children with stuttering investigated in other international studies. Regarding investigations about MMN, there were only two international studies with a number higher than ours – one with 18 children,^(7)^ and another with 12 children.^(14)^ Regarding studies about P300, there was one Brazilian study that included 13 children who stuttered.^(9)^ It is known that an elevated number of individuals can ensure more statistical power to a study; however, studies developed with groups of stuttering patients and Control Groups present a reduced number of children in their sample – less than 20.^(7,9,14)^

In the CG, there was equivalence between the number of boys and girls, but, in the SG, the percentage of boys was much higher than of girls, which was expected. The fact that girls develop language earlier than boys may contribute to this difference^(15)^

In the area of auditory electrophysiology, the magnitude (amplitude) and the speed (latency) of the processing reflect the efficacy of neural functions.^(14)^ In this study with MMN, through a tone burst stimulus, with electrodes placed at Fpz, delayed latency was found in relation to the CG. This delay suggests that the SG required more time to differentiate the standard from the rare stimuli.^(3)^ Delayed latency suggests an altered central auditory processing.^(12)^

Neuroimaging has shown that a deficit in auditory processing may stem from a deficit in the temporal processing of information.^(16)^ In addition to PDS, latency delays were shown in children with specific language impairment (SLI)^(17)^ and dyslexia.^(18)^

Contrary to our findings, there are reports in the literature of lower amplitude values of MMN in children with stuttering in comparison to the Control Group.^(14)^ The explanation offered by the researchers was of an inefficient neural processing of the differences between speech sounds. The stimulus used was linguist and not pure tone. These results suggest generalized central difficulties in sound differentiation,^(14)^ and imprecision in auditory discrimination skills.^(3)^

Still on the amplitude of MMN, our results showed significant differences between the investigated groups, with increased amplitude in the SG. Although a great part of clinical studies present reduced or even absent MMN amplitude,^(3)^ results similar to ours were reported in the literature about studies on stuttering, but with different protocols from ours and with speech stimuli – in this case, phoneme variation.^(19)^ Such results were attributed to a difficulty to synchronize the neural activities of auditory areas, leading to an exacerbated response.^(19)^ The visible increase in response, which generates a greater amplitude of the wave, may be related to the also exacerbated quantity of neurons recruited for the resolution of the task. Functional neuroimaging shows that brain areas that are more activated indicate greater difficulty and cognitive demand to complete a task.^(20)^ However, this finding is not a marker for a specific type of disorder, but it may suggest an altered cognitive standard and be helpful as a risk indicator.^(3)^

Regarding the P300, the SG obtained latencies that were significantly more delayed than the CG. Findings of young adults with stuttering also corroborate our data on the latency of P300,^(21)^ showing reduced auditory attention.^(22)^ In one of the studies, in addition to P300, there was a behavioral evaluation of temporal processing using the Random Gap Detection Test (RGDT). It was found that the latency delay of P300 and the low performance on the RGDT affect the speed of auditory processing of sound.

In addition to stuttering, other populations presented delayed latencies in P300, such as children with Down syndrome.^(23)^ Based on that, it seems well documented that latency delays of the P300 wave can suggest alterations in the processing of auditory information. The amplitude of P300 in the SG was reduced in comparison to the CG. It is possible that some individuals in the SG presented a deficit in non-linguistic auditory processing, which may be related to an alteration in cortical processing.^(24)^ Similar results in the amplitude of P300 were found in young adults who stutter.^(25)^

The morphology of the waves of the children in the SG was altered in MMN and P300. In individuals with auditory thresholds within normality, a more defined morphology of the waves is expected starting at the age of 8 years.^(26)^ In this study, there were a few children in the SG who were 6 years old, which could explain this finding. However, this hypothesis may be controversial, because the CG also included 6-year-old children and the group presented a more defined morphology. Therefore, there seems to be a relation between stuttering and the alterations in the morphology of the waves of the investigated LLAEP. Children with no auditory complaints, but with complaints regarding learning difficulties, also presented alterations in the morphology of the P300 wave.^(27)^

Regarding manual preference, the SG presented a higher rate of left-handed children than the CG. In typical development, even with some controversies, right-handed children have performed better at tests that include speech motor skills and cognitive language tests. This advantage suggests left hemispheric laterality for motor and speech processing.^(28)^ The left hemisphere is responsible for the linguistic analysis of sound, and the right hemisphere is responsible for the encoding of non-linguistic sounds, such as musical rhythms.^(29)^ Therefore, it was expected that left-handed and right-handed children would present differences in their performances on the same test. Our results corroborate this hypothesis, partly because in MMN and P300 left-handed children showed more latency delay than right-handed children, even though there was no significant difference between the groups. The amplitude of the left-handed children in relation to the right-handed children were greater in MMN and equal in P300. The low number of children in each group may have affected our statistical results and a higher number of children may yield different results. However, similar results were found in the literature.^(4)^

## CONCLUSION

There is a significant latency delay of the long latency auditory evoked potentials Mismatch Negativity and P300 cognitive potential of the children with stuttering in comparison to children who do not stutter.
