# Supplementary material for: Long-latency auditory evoked potential in children with stuttering
Source: Einstein (Sao Paulo). 2020 Jun 3;18:eAO5225. doi: 10.31744/einstein_journal/2020AO5225 (PMC7279890; doi:10.31744/einstein_journal/2020AO5225)
Supplement: Translation

# Translation: Potenciais evocados auditivos de longa latência em crianças com gagueira

**Authors:** Gislaine Machado Jerônimo, Ana Paula Rigatti Scherer, Pricila Sleifer

**Affiliations:** 1 Brasil Universidade Federal do Rio Grande do Sul, Porto Alegre, RS, Brasil.

**Keywords:** Eletrofisiologia, Potenciais evocados, Audição, Gagueira, Criança

## Abstract

**Objetivo:**

Analisar os valores de latência e amplitude do *Mismatch Negativity* e potencial cognitivo P300 em crianças com gagueira, sem queixas auditivas, com limiares auditivos dentro dos padrões de normalidade, comparando aos achados de um Grupo Controle.

**Métodos:**

Estudo transversal, do qual participaram 50 crianças de ambos os sexos, sendo 15 com gagueira e 35 sem gagueira, entre 6 e 11 anos de idade, sem diagnóstico de patologias otológicas ou outras doenças. Todas as crianças realizaram avaliação audiológica periférica (meatoscopia, audiometria tonal, audiometria vocal e medidas de imitância acústica) e avaliação audiológica central (*Mismatch Negativity* e potencial cognitivo P300). Para avaliação da fluência, as crianças com gagueira realizaram anamnese específica, seguida da filmagem de uma fala espontânea, que foi transcrita e analisada quanto à severidade da gagueira.

**Resultados:**

Houve diferença significativa nas latências do *Mismatch Negativity* e do potencial cognitivo P300, assim como na amplitude do *Mismatch Negativity*.

**Conclusão:**

Verificou-se atraso nas latências do *Mismatch Negativity* e potencial cognitivo P300 nas crianças com gagueira, assim como aumento na amplitude do *Mismatch Negativity*, ao serem comparados com crianças do Grupo Controle. No Grupo com Gagueira foram igualmente identificadas alterações na morfologia das ondas.

## INTRODUÇÃO

Os potenciais evocados auditivos de longa latência (PEALL) são utilizados na investigação cognitiva, com destaque para o potencial cognitivo P300, um potencial endógeno, o qual mostra respostas bioelétricas que percorrem o tálamo e o córtex.^(1,2)^ O *Mismatch Negativity* (MMN), outro potencial endógeno, é um PEALL que reflete a resposta cerebral elétrica das habilidades de processamento, discriminação e memória auditiva.^(3)^ Ele é um potencial que detecta a mudança de estímulo auditivo discriminável, tendo por sítios geradores o córtex frontal, o tálamo e o hipocampo.^(3,4,5)^

A principal característica do MMN é seu registro sem a influência da atenção do sujeito e nem exigências de tarefas, tornando-o eficaz para estudos clínicos em diferentes populações.^(6)^Já o P300 é gerado voluntariamente, de forma ativa na resolução de uma tarefa específica; avalia as habilidades de percepção, atenção e memória auditiva. Seus sítios geradores são o hipocampo, a formação reticular mesencefálica, o tálamo medial, o córtex pré-frontal e as áreas de associação parietotemporal.^(1)^

Na população infantil, há estudos com MMN^(3,7,8)^ em populações com transtorno do espectro autista, transtornos do processamento auditivo, fissura labiopalatina, prematuridade e transtorno do desenvolvimento da linguagem. A maioria dos artigos são sobre dislexia, sendo o MMN pouco explorado em indivíduos com gagueira.^(8)^Na população brasileira, foi encontrado apenas um estudo publicado com P300 em crianças com gagueira.^(9)^ Até o presente momento, são desconhecidos estudos nessa população com ambas as técnicas, MMN e P300, utilizadas concomitantemente.

A gagueira é um distúrbio da fluência, que envolve alterações no fluxo da fala. São interrupções involuntárias e atípicas, dificultando uma fala fluída e suave, caracterizada por repetições, prolongamentos e bloqueios. Sua prevalência é de 1% da população mundial adulta e de até 5% na população infantil.^(10,11)^ A gagueira iniciada na infância é chamada de gagueira desenvolvimental persistente (GDP) e tem incidência de 5 para cada 100 crianças.^(12,13)^

Teorias atuais incorporam a união de fatores genéticos, neurológicos, motores, linguísticos e ambientais para explicar a etiologia da GDP.^(14)^ A pesquisa com neuroimagem evidencia que crianças que gaguejam possuem diferenças neuroanatômicas e funcionais.^(10)^

Aspectos auditivos podem estar prejudicados e interferir na fluência da fala.^(11)^ Assim, a pesquisa dos PEALL para a área de gagueira é de grande relevância, pois pode auxiliar em uma melhor compreensão dos fatores que interferem no desempenho da fluência de fala, por meio de avaliação objetiva, bem como auxiliar em técnicas terapêuticas de reabilitação.

## OBJETIVO

Analisar os valores de latência e amplitude dos potenciais evocados auditivos de longa latência, em crianças com gagueira, sem queixas auditivas, com limiares auditivos dentro dos padrões de normalidade, comparando aos achados aos de um Grupo Controle.

## MÉTODOS

Estudo realizado ao longo do ano de 2017, observacional, transversal e comparativo, contemporâneo e individual, aprovado pelo Comitê de Ética em Pesquisa do Instituto de Psicologia da Universidade Federal do Rio Grande do Sul (UFRGS), sob o número 2011039. A amostra por conveniência consistiu de crianças de ambos os sexos, entre 6 e 11 anos de idade, divididas em dois grupos: Grupo Estudo com Gagueira Desenvolvimental (GE), composto por crianças com severidade de leve a muito grave, e Grupo Controle (GC), composto por crianças com desenvolvimento normal.

As informações sobre idade, sexo, preferência manual, escolaridade, comprometimento linguístico, social, neural e otológico, assim como ter ou não frequentado terapia fonoaudiológica, foram obtidas por anamnese geral. Como critério de exclusão, foram consideradas queixas auditivas, de linguagem ou de aprendizagem, não saber contar de 1 a 50, qualquer tipo de perda auditiva, avaliação otorrinolaringológica alterada, apresentação de síndromes ou alterações psiquiátricas, ter participação em terapia fonoaudiológica para gagueira e, por algum motivo, não ter realizado os procedimentos e nem concluído os exames. Todas as crianças realizaram avaliação auditiva periférica e avaliação eletrofisiológica central.

O GE foi previamente triado por duas fonoaudiólogas. O diagnóstico de gagueira foi verificado e confirmado por profissionais fonoaudiólogos com experiência na área. Referente à análise de concordância sobre o diagnóstico, foi utilizada a medida de Kappa.

Para avaliação audiológica, foram realizadas anamnese, inspeção dos meatos auditivos externos, audiometria tonal limiar, logoaudiometria e imitanciometria. A pesquisa do MMN e P300 foi realizada por equipamento Masbe ATC Plus, da marca Contronic^®^, com uso de fone de inspeção earfone 3A e eletrodos de prata. A impedância elétrica foi inferior a 5Ω em cada derivação, e a diferença entre os três eletrodos não excedeu a 2Ω. Após a verificação da impedância, foi realizada a varredura do eletroencefalograma.

Os parâmetros utilizados para a pesquisa do MMN foram estímulos auditivos apresentados de modo monoaural, com frequência de 1.000Hz (50 ciclos), para o estímulo frequente, e 2.000Hz (50 ciclos), para o estímulo raro, em uma intensidade de 70 dBNA para ambos. A taxa de apresentação foi de 1,8 pulso por segundo (pps). As promediações foram de 2.000 estímulos e o paradigma de 90/10, com polaridade alternada. Na aquisição, o fundo de escala foi de 200µV, filtro passa-alta de 1Hz, filtro passa-baixa de 20Hz, Notch – SIM, janela temporal 500ms e amplitude do traçado até 7,5µV. Durante esse processo, os indivíduos foram condicionados a assistirem um vídeo interessante e silencioso no *tablet*, com a intenção de desviar a atenção sobre os estímulos auditivos apresentados. Antes de iniciar o exame, a criança foi orientada sobre a execução do teste, no sentido de prestar atenção no vídeo.

Na pesquisa do P300, foram utilizados estímulos binaurais com intensidade de 80 dBNA para ambas as orelhas. A frequência foi de 1.000Hz com 50 ciclos de duração e 20% de *rise* e *decay time*, com envelope trapezoidal, e a do estímulo raro foi *tone burst* de 2.000Hz, com 100 ciclos de duração com 20% de *rise* e *decay time* com envelope trapezoidal, apresentados em paradigma do tipo raro-frequente (*odd ball*), com probabilidade de 80% e 20% de aparecimento, respectivamente. Os estímulos foram apresentados na taxa de 0,8pps. Na aquisição, o fundo de escala foi de 200 µV, filtro passa-alta de 01Hz, filtro passa-baixa de 20Hz, Notch – SIM, e janela de leitura utilizada foi de 1.000ms. Durante este processo, as crianças tiveram que prestar atenção nos estímulos auditivos frequentes e raros apresentados, contando apenas os raros. A latência do P300 foi marcada no ponto de máxima amplitude da onda, e sua análise foi realizada por meio da onda resultante.

Os exames foram realizados duas vezes. Com objetivo de verificar a concordância da análise do MMN e P300, foram utilizados os métodos estatísticos de Kappa. Os resultados foram organizados sob forma de estatística descritiva. O teste Kolmogorov-Smirnov foi utilizado para avaliar a normalidade dos dados. Para comparar as orelhas em relação aos resultados de latência e amplitude, o teste *t* Student foi aplicado. O nível de significância adotado foi de 5% (p<0,05), e as análises foram realizadas pelo software (SPSS) para Windows, versão 17.0.

## RESULTADOS

Foram selecionadas 15 crianças com gagueira, as quais tinham diferentes graus de severidade: 4 de leve para moderada, 6 moderada, 2 moderada para grave, 1 grave e 2 grave para muito grave. Ao total, 50 crianças participaram efetivamente do estudo. A tabela 1 apresenta a caracterização da amostra.

**Tabela 1:** Caracterização da amostra

Variáveis	GE (n=15)	GC (n=35)
Sexo		
Masculino	12 (80)	20 (57,14)
Feminino	3 (20)	15 (42,86)
Preferência manual		
Destro	8 (53,3)	31 (88,6)
Canhoto	7 (46,7)	3 (8,6)
Ambidestro	0	1 (2,9)
Idade, mínimo/máximo (6-11)	8,40±1,80	9,29±1,52

Resultados expressos por n (%) ou média±desvio padrão.

GE: Grupo Estudo com Gagueira Desenvolvimental; GC: Grupo Controle.

Houve excelente concordância entre juízes na análise da severidade da gagueira (Kappa 0,82), bem como na análise dos componentes dos PEALL MMN e P300 (Kappa 0,89). De acordo com o coeficiente de correlação interclasse, obtiveram-se 0,76 para o MMN e 0,85 para o P300, com correlação quase perfeita.

Todas as crianças apresentaram resposta na avaliação do MMN. As médias e os desvio padrão da latência e amplitude, tanto da orelha direita (OD) como da orelha esquerda (OE), estão apresentadas na tabela 2.

**Tabela 2:** Avaliação do *Mismatch Negativity* para latência e amplitude

Orelha	GE (n=15)		GC (n=35)		Valor de p^*^
	
	n	Média±desvio padrão	n	Média±desvio padrão	
Direita					
Latência MMN	15	332,01±77,65	35	185,24±43,57	<0,001^*^
Amplitude MMN	15	8,11±3,28	35	5,25±1,61	<0,001^*^
Esquerda					
Latência MMN	15	330,66±81,21	35	182,24±37,80	<0,001^*^
Amplitude MMN	15	7,75±3,30	35	5,65±2,21	0,011^*^

* Teste *t* de Student; p≤0,05 significativo.

GE: Grupo Estudo com Gagueira Desenvolvimental; GC: Grupo Controle; MMN: *Mismatch Negativity.*

Na pesquisa de P300, foi observada resposta em 14 das crianças com gagueira, com exceção de 1. As diferenças de desempenho observadas entre os grupos estão ilustradas na tabela 3.

**Tabela 3:** Resultados obtidos na avaliação do P300 para latência e amplitude

Variáveis	GE (n=14)		GC (n=35)		Valor de p^*^
	
	n	Média±desvio padrão	n	Média±desvio padrão	
Latência P300	14	697,19±142,84	35	308,17±18,81	<0,001^*^
Amplitude P300	14	11,70±3,89	35	13,53±4,85	0,216

* Teste *t* de Student; p≤0,05 significativo.

GE: Grupo Estudo com Gagueira Desenvolvimental.

Na tabela 4, estão evidenciadas as médias e os desvio padrão da latência e da amplitude dos PEALL MMN e P300 das crianças canhotas em relação às destras.

**Tabela 4:** Comparação da latência e da amplitude do *Mismatch Negativity* e P300, conforme preferência manual das crianças do Grupo Estudo com Gagueira Desenvolvimental

Variáveis	Destras		Canhotas		Valor de p^*^
	
	n	Média±desvio padrão	n	Média±desvio padrão	
MMN orelha direita					
Latência	8	322,40±85,83	7	342,99±72,17	0,880
Amplitude	8	7,99±3,18	7	8,26±3,63	0,627
MMN orelha esquerda					
Latência	8	314,04±74,85	7	349,65±89,84	0,065
Amplitude	8	6,30±2,62	7	9,42±3,37	0,417
P300					
Latência	8	706,30±147,12	6	685,05±149,80	0,997
Amplitude	8	11,71±3,14	6	11,71±5,06	0,795

* Teste *t* de Student; p≤0,05 significativo.

MMN: *Mismatch Negativity.*

## DISCUSSÃO

A amostra analisada é compatível com o número de crianças com gagueira investigadas em outros estudos internacionais. Na pesquisa com MMN, superior ao nosso número, foi encontrado apenas um estudo internacional, com 18 crianças;^(7)^ outro contou com 12 crianças.^(14)^ Na pesquisa com P300, um estudo nacional investigou 13 crianças com gagueira.^(9)^ Sabe-se que um número elevado de sujeitos pode garantir maior força estatística ao estudo, entretanto estudos desenvolvidos com gagueira e Grupos Controles apresentam número reduzido de crianças em sua amostra − este número é inferior a 20.^(7,9,14)^

No GC, houve equivalência entre os números de meninos e meninas, porém, no GE, o percentual de meninos foi bem maior do que o de meninas, o que já era esperado. O fato de as meninas desenvolverem a linguagem mais cedo pode contribuir para esta diferença.^(15)^

Na área da eletrofisiologia auditiva, a magnitude (amplitude) e a velocidade (latência) do processamento refletem a eficácia das funções neurais.^(14)^ Na presente pesquisa com MMN, por meio do estímulo *tone burst*, com eletrodos em posição Fpz, foram evidenciadas latências tardias no GE, em relação ao GC. Este atraso indica que o GE necessitou de mais tempo para diferenciar o estímulo padrão do estímulo raro.^(3)^ A latência tardia sugere processamento auditivo central alterado.^(12)^

A neuroimagem tem mostrado que *deficit* no processamento auditivo podem ser decorrentes de *deficit* no processamento temporal da informação.^(16)^ Além da GDP, atrasos na latência foram evidenciados em crianças com *deficit* específico de linguagem (DEL)^(17)^ e dislexia.^(18)^

Contrário aos nossos achados, há relato na literatura de valores de amplitude do MMN menores em crianças com gagueira comparadas a um Grupo Controle.^(14)^ A explicação dos pesquisadores foi de um processamento neural ineficaz das diferenças entre os sons de fala. O estímulo utilizado foi linguístico e não tom puro. Estes resultados sugerem dificuldades centrais generalizadas na diferenciação do som,^(14)^ bem como uma imprecisão na habilidade de discriminação auditiva.^(3)^

Ainda sobre a amplitude do MMN, nossos resultados mostraram diferenças significativas entre os grupos investigados, em favor de uma amplitude aumentada no GE. Embora grande parte dos estudos clínicos apresente amplitudes do MMN reduzidas ou mesmo ausentes,^(3)^ resultados semelhantes aos nossos foram reportados na literatura com gagueira, mas com protocolos diferentes aos do atual estudo e com estímulos de fala − no caso, variação de fonemas.^(19)^ Tais resultados foram atribuídos a uma dificuldade de sincronização da atividade neural das áreas auditivas, ocasionando resposta exacerbada.^(19)^ O aumento visível na resposta, o qual gera amplitude maior da onda, pode estar relacionado à quantidade também exagerada de neurônios recrutados para resolução da tarefa. A neuroimagem funcional mostra que áreas cerebrais mais ativadas indicam maior dificuldade e demanda cognitiva para dar conta de uma tarefa.^(20)^ No entanto, este achado não é um marcador de um tipo específico de desordem, mas pode indicar um padrão cognitivo alterado e ser útil como indicador de risco.^(3)^

Atinente ao P300, o GE obteve latências significativamente mais atrasadas do que o GC. Achados de adultos jovens com gagueira igualmente corroboram nossos dados de latência do P300,^(21)^ sendo evidenciada atenção auditiva reduzida.^(22)^ Em um dos estudos, além do P300, foi realizada avaliação comportamental do processamento temporal, por meio do Teste de Detecção de Intervalos Aleatórios (RGDT - *Random Gap Detection Test*). Foi verificado que o atraso na latência do P300, bem como o baixo desempenho no RGDT, impactam na velocidade de processamento auditivo do som.

Além da gagueira, outras populações apresentaram latências atrasadas no P300 como, por exemplo, crianças com síndrome de Down.^(23)^Com base no exposto, parece bem documentado que o atraso na latência da onda P300 pode fornecer indícios de alterações no processamento da informação auditiva.

A amplitude do P300 no GE foi reduzida, na comparação com o GC. Há a possibilidade de uma parcela do GE exibir *deficit* no processamento auditivo não linguístico, e estes estarem relacionados a uma alteração do processamento cortical.^(24)^ Resultados semelhantes na amplitude do P300 foram encontrados em adultos jovens com gagueira.^(25)^

Tanto no MMN como no P300, a morfologia das ondas das crianças do GE mostrou-se alterada. Em indivíduos com audição dentro dos padrões de normalidade, espera-se morfologia das ondas mais definida a partir dos 8 anos de idade.^(26)^ No presente estudo, havia algumas crianças no GE com 6 anos, o que poderia justificar este achado. Contudo, esta hipótese pode ser questionada, uma vez que, no GC, também tinha crianças com 6 anos, e o grupo apresentou morfologia mais definida. Assim, parece haver relação entre a gagueira e as alterações na morfologia da onda dos PEALL investigados. Crianças sem queixas auditivas, mas com queixas de dificuldade de aprendizagem, também apresentaram alterações na morfologia da onda P300.^(27)^

Referente à preferência manual, o GE apresentou percentual de canhotos maior do que o GC. No desenvolvimento típico, mesmo com algumas controvérsias, crianças com preferência manual direita têm apresentado melhor desempenho em testes que incluem habilidade motora da fala e em testes cognitivos de linguagem. Esta vantagem sugere lateralidade hemisférica esquerda para o processamento motor e de fala.^(28)^ O hemisfério esquerdo seria responsável pela análise linguística do som, ao passo que o hemisfério direito seria responsável pela codificação de sons não linguísticos, como ritmo musical.^(29)^ Desse modo, esperava-se que crianças canhotas e destras apresentassem diferenças de desempenho em um mesmo teste. Nossos resultados corroboram esta hipótese, em parte, pois, no MMN e P300, embora não tenha ocorrido diferença significativa entre os grupos, as crianças canhotas obtiveram latências mais atrasadas do que as destras. As amplitudes dos canhotos em relação aos destros foram maiores no MMN e iguais no P300. O número pequeno de crianças em cada um dos grupos pode ter interferido nos resultados estatísticos e um número maior de crianças poderia alterar os resultados. Entretanto, resultados semelhantes foram evidenciados na literatura.^(4)^

## CONCLUSÃO

Existe atraso significativo nas latências dos potenciais evocados auditivos de longa latência *Mismatch Negativity* e potencial cognitivo P300 das crianças com gagueira, ao serem comparados com crianças sem este tipo de acometimento.
